# Pan-Genome Analysis of *Cannabis sativa*: Insights on Genomic Diversity, Evolution, and Environment Adaption

**DOI:** 10.3390/ijms26178354

**Published:** 2025-08-28

**Authors:** Shuyu Wang, Xue Zhong, Yuhui Cheng, Ying Yu, Jifeng Wan, Qingqing Liu, Yongjun Shu, Xiuju Wu, Yong Li

**Affiliations:** 1 College of Life Sciences, Northeast Agricultural University, Harbin 150030, China; 2 Key Laboratory of Molecular Cytogenetics and Genetic Breeding of Heilongjiang Province, College of Life Science and Technology, Harbin Normal University, Harbin 150025, China; shuyj@hrbnu.edu.cn

**Keywords:** *Cannabis sativa*, pan-genome, PAV, flexible genes, gene loss, stress resistance, cannabis breeding

## Abstract

*Cannabis sativa* is a crop which has been cultivated since ancient times, with important cultural and industrial value. Due to its substantial economic impact, cannabis has attracted widespread scientific attention. A pan-genome is a significant tool for breeding, because it provides a comprehensive representation of genetic diversity. To provide a valuable tool for Cannabis breeding, we constructed a Cannabis pan-genome based on 113 accessions. A total of 24,679,380 bp of non-reference-genome sequences were assembled, identifying 1313 protein-coding genes. Using pan-genome analyses, a total of 32,428 gene presence/absence variations (PAVs) were obtained, and gene loss was recovered during the domestication of Cannabis. By partitioning the pan-genome using PAVs, a total of 23,309 core genes were identified, accounting for 71.88% of all genes in the pan-genome. In particular, there were 7148 flexible genes, making up 22.05% of the pan-genome. The flexible genes were associated with adaptive traits, including stress resistance and disease resistance in Cannabis. Population genetic analysis presented gene distribution, gene flow, and gene specificity on a pan-genome level. These results provide important genetic basis, functional genes, and guidance for Cannabis breeding.

## 1. Introduction

Cannabis (*Cannabis sativa*, 2n = 20) is an annual erect herbaceous plant in the Cannabaceae family and Cannabis genus, with a long history of cultivation worldwide [1]. As a cultivated species, Cannabis has nine pairs of autosomes and one pair of sex chromosomes, with both dioecious and monoecious varieties. Cannabis plays a significant role in cultural, daily life, and industrial applications [2]. Archeological evidence also confirms the versatility of Cannabis in ancient times. Chinese archeologists have detected cannabinol-related residues at a historic site on a mountaintop in western China. The presence of these Cannabis residues suggests that Cannabis was probably used by the ancient people of this area for rituals or as a medicine [3]. The different tissues of Cannabis have a wide range of industrial and medical applications. Its bast fibers, for example, are used in the manufacture of textiles, while the seeds are rich in oil, which can be processed into edible oils and nutritional supplements. In addition, the glandular hairs on the inflorescences, leaves, and bracts of *Cannabis sativa* are rich in cannabinoid compounds, which are of great medical value in the field of pharmaceuticals. With the development of pharmacological research, tetrahydrocannabinol (THC) has been found in Cannabis, which is a compound with strong psychoactive properties that is addictive when taken for a long period of time, which is why Cannabis has been classified as a drug in some countries around the world. The THC content is currently the main criterion for determining the type of Cannabis; Cannabis varieties with more than 0.3% THC are classified as drug type (marijuana) on a dry weight basis, and Cannabis varieties with less than 0.3% THC are classified as industrial type (hemp). Subsequent research on cannabinoids led to the discovery of another cannabinoid, cannabidiol (CBD), which is widely used clinically for pain relief, depression, and psychiatric disorders [4]. CBD also has anti-tumor properties that may improve median survival time in patients with advanced tumors along with treatments such as pain relief [5]. In addition, CBD has the effect of treating acne and inhibiting sebum secretion, so CBD is also used to add to cosmetics [6].

Several genomes of Cannabis have been published [7,8,9]. A pan-genome refers to a non-redundant collection of genetic information contained in all individuals of a certain species. A pan-genome can meet the comprehensive mutation detection of a species or even all species in a genus, and can identify new genes to answer problems that cannot be solved by a single reference genome. A pan-genome enables the discovery of previously hidden genetic variations, provides insights into crop domestication and breeding, and enables the identification of functional genes [10]. Based on the pan-genome, more variants can be detected and analyzed, such as the detection of gene presence/absence variants (PAVs). Compared with other variations, PAVs have a more profound impact on the genome, because they not only present the structure of the genome, but also directly reveal the function and traits of genes [11,12,13,14,15,16,17,18,19].

Cannabis has important value in industry, food, and medicine. In particular, since many countries have changed their Cannabis policy to increase its value [20], breeding new varieties will be in demand. A pan-genome is a valuable tool to improve breeding. To construct a Cannabis pan-genome and provide a genetic resource for Cannabis breeding, we sequenced the genomes of three Cannabis varieties from Heilongjiang Province, China, and constructed a pan-genome by integrating these sequences with 110 resequencing datasets and the CBDRx reference genome [9]. We obtained 24,679,380 bp of de-redundant non-reference genome sequences, and identified 1313 protein-coding genes. Based on this pan-genome, several key findings emerged, such as gene loss during Cannabis domestication and the association of dispensable genes with adaptive traits, including stress and disease resistance.

## 2. Results

### 2.1. Assembly of Cannabis Pan-Genome and Gene Prediction

**Figure 1:**
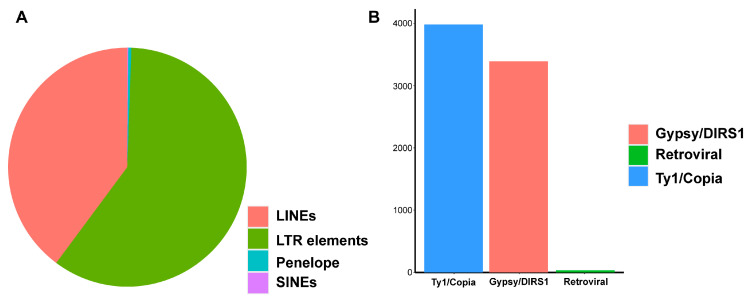
Retroelements in non-reference sequences. (**A**) Retroelement distribution. (**B**) LTR element distribution.

The resequencing data of 113 Cannabis varieties are mapped to the reference genome, and a total of 2.04 Gbp of non-reference sequences are obtained. After removing redundant sequences, 14,306 contigs are obtained, with a total length of 24,679,380 bp. These non-reference sequences are combined with reference genome CBDRx as the Cannabis pan-genome. Using RepeatModeler and RepeatMasker to predict repetitive sequences in non-reference sequences, finally, a total of 12,636,404 bp of repetitive sequences are masked, accounting for 51.20% of all non-reference sequences. This is lower than reference genome CBDRx (63%). The largest proportion of repetitive sequences is retroelements, accounting for 32.74% (Table 1). Among them, long interspersed nuclear elements (LINEs) and long terminal repeats (LTRs) are the largest proportions (Figure 1). In contrast, the Cannabis reference genome has few LINEs (~0.13%). LINEs can result in nonallelic homologous recombination (NAHR) to generate long indels [21,22,23]; this may explain why non-reference sequences are absent from the reference genome.

Using previously assembled Cannabis transcript data as transcript evidence, and using homologous protein sequences to assist gene prediction, 1313 new protein-coding genes were identified in the non-reference sequence (Table 2) and combined with genes from the reference genome for subsequent analysis.

After gene prediction, we used multiple databases to annotate all proteins in the pan-genome. More than 99.1% of proteins were annotated using Uniref50 and other databases (Table 3). A total of 1274 proteins from non-reference sequences were annotated, accounting for 97.03% of all non-reference genes. The high percentages of annotated proteins also indicated that all these proteins were reliable. GO (Gene Ontology) and KEGG (Kyoto Encyclopedia of Genes and Genomes) annotations were also carried out (Table 3), and all these annotations were used for subsequent analysis.

### 2.2. Population Genetic Analysis Based on Pan-Genome

**Figure 2:**
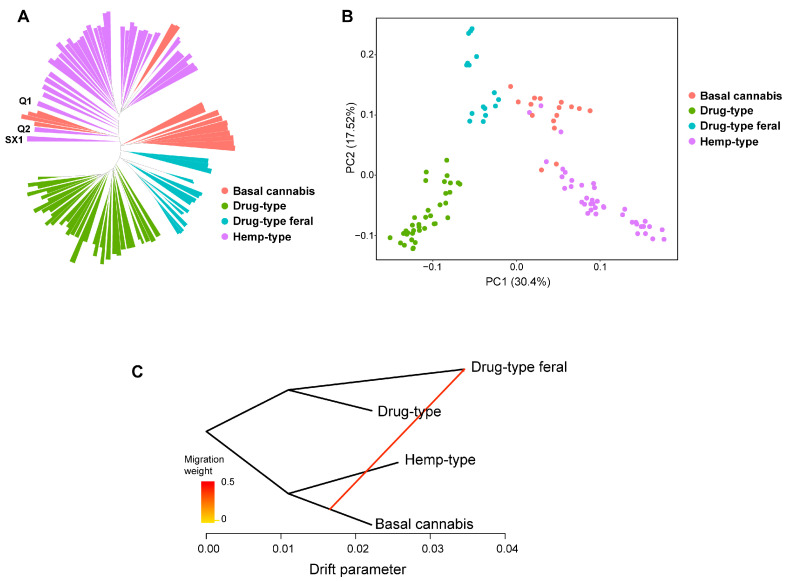
Population structure and gene flow of Cannabis accessions. (**A**) Neighbor-joining phylogenetic tree based on SNPs. Samples clustered by Cannabis groups. (**B**) Principal component analysis based on SNPs. Different colors represent different Cannabis groups, and shapes represent variety categories. (**C**) Gene flow analysis based on SNPs, completed by Treemix; the arrow represents the direction of gene flow. Gene exchange present in the feral drug-type Cannabis group and basal Cannabis group.

After mapping the Cannabis pan-genome, we kept 109 Cannabis accessions for subsequent analysis. We identified 13,905,272 single nucleotide polymorphisms (SNPs) for population genetic analysis. The phylogenetic tree was constructed using the same strategy as in the previous study [24]. Principal component analysis and gene flow analysis were also performed based on SNPs. The samples are obviously clustered into four parts (Figure 2A,B), which is similar to reported results [9]. Drug-type Cannabis and feral drug-type Cannabis are on the same branch, showing the clustering phenomenon of their respective groups. This result also indicates that the pan-genome is reliable and can reflect evolution relation. The hemp-type and the basal Cannabis (naturally cultivated varieties or landraces) are on the same branch; Qing2 (Q2) and NEB1, NEB2, NEB3, and NEB4 belonging to the basal Cannabis are on the same branch; and the FKS and FNA belonging to the basal cannabis are clustered into the hemp-type group.

The gene flow ranges from the base Cannabis population to the feral drug-type Cannabis population. In the phylogenetic tree and principal component analysis (PCA), the feral drug-type Cannabis group is closer to the basal Cannabis group, which indicates gene exchange happened between the two groups (Figure 2C). This result indicates few gene exchanges happened among different types of Cannabis, and shows great potential to gain new genotypes and traits for breeding.

To analyze the selection interval during domestication in the pan-genome, XP-CLR and Fst were used to identify the selection signal, and the first 10% of the results were used to identify the selection interval. There were 1182 genes in all selection intervals between the feral varieties of basal Cannabis and hemp-type Cannabis cultivars, which contained 1172 reference genome genes and 10 non-reference sequence genes. There were 537 genes in all intervals between the feral drug-type Cannabis and drug-type Cannabis populations, including 534 reference genome genes and 3 non-reference genome genes. The two groups of the selection interval shared 45 genes, 44 of which were from the reference genome and 1 was from the non-reference genome.

**Figure 3:**
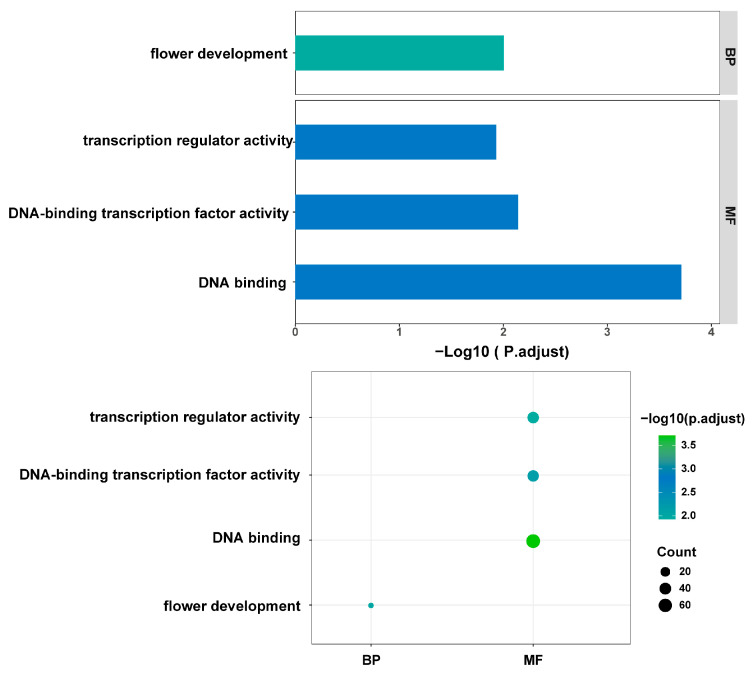
Analysis of GO enrichment of genes in the selection interval of domestication of hemp-type Cannabis. These genes are involved in gene regulation and flowering and should be associated with agronomic traits. BP: biological process, MF: molecular function.

To study the functional role of genes in the selection intervals, functional enrichment of genes was performed. Significant enrichment was observed in the selection interval between the feral population of basal Cannabis and the cultivated population of hemp-type Cannabis (adjusted *p* value < 0.05). Among them, in terms of biological processes (BPs), these genes are mainly enriched in the entry “Flower development”. In terms of molecular function (MF), the enriched entries are “DNA binding”, “DNA-binding transcription factor activity”, and “transcription regulator activity” (Figure 3). This result discovers the genes and their functions associated with domestication. In the selection interval between the feral drug-type Cannabis group and drug-type Cannabis group, neither the GO enrichment nor the KEGG enrichment have significant entry.

A large number of genes related to the regulation of flowering have been found in the selected genes of the hemp-type population, such as *Hd3a, HDR1*, and *FPF1*. Fiber-related genes have also been found, such as *KCS, GA20-oxidase1*, and *TBL*. KCS affects the elongation of fibers in cotton; GA20-oxidase1 increases plant height, fiber length, and strength after overexpression; and TBL affects the thickening and strength of cell wall, which may also be related to fiber growth [25,26,27]. Genes that are in the non-reference genome are also involved in selection, and they are mostly related to methylation, growth and development, and stress response. These genes were selected perhaps because people wanted to obtain more fiber in hemp-type Cannabis. In the selection interval of drug-type Cannabis, there are a large number of genes related to substance metabolism. Similarly to industrial hemp, there are genes that regulate flowering (such as *SPL*, *LATE*, etc.), and there is also the *Seipin-1* gene, which is involved in the biosynthesis of lipid droplets to promote pollen transmission and reduce seed dormancy. The *SPL* gene has been reported to be involved in glandular hair growth in *Artemisia japonica* and tillering in switchgrass [28,29,30,31]. This gene has been selected in drug-type Cannabis, because cannabinoids are synthesized and stored in large quantities in glandular hairs. This result is consistent with the fact that the cultivation direction of drug-type Cannabis is mainly to grow more branches and glandular hairs to increase the content of cannabinoids.

### 2.3. PAV Analysis of Cannabis Pan-Genome

**Figure 4:**
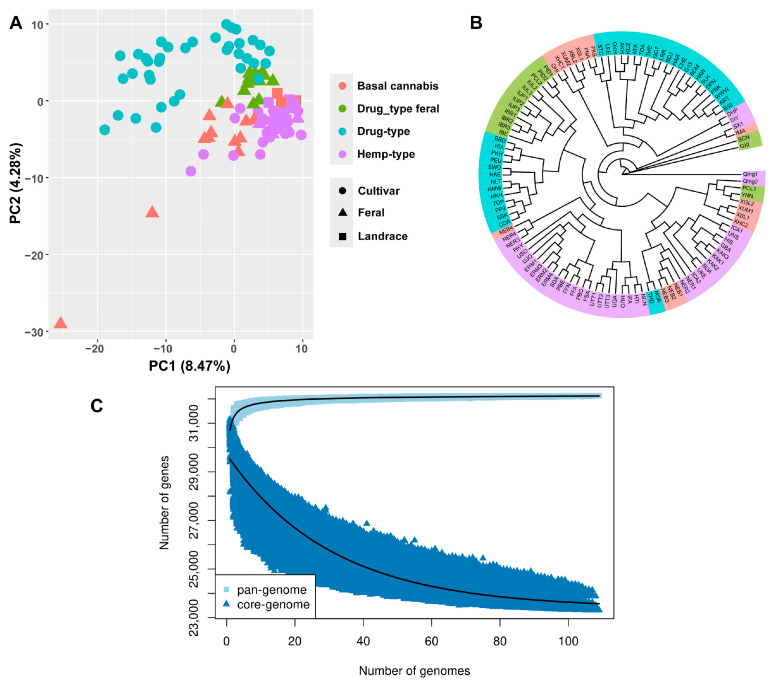
Phylogenetic analysis and principal component analysis based on PAVs. (**A**) Principal component analysis based on SNPs. Different colors represent different Cannabis groups, and shapes represent variety categories. Samples from same groups clustered together. (**B**) Maximum likelihood phylogenetic tree based on PAVs. Samples from same groups are present in same branches, which is similar to phylogenetic tree based on SNPs. This reflects that PAVs are reliable. (**C**) Changes in pan-genome and core genome size with sample filling. Gene numbers of both core genome and pan-genome changed slowly when genome number was close to sample number used in this study. This indicates sample number in this study is enough to construct reliable pan-genome.

All 32,428 genes of the pan-genome and resequencing data of 109 samples were used for PAV analysis. PCA based on PAVs showed that the samples clustered according to their categories, such as the hemp type and drug type (Figure 4A). This is consistent with the PCA based on SNPs. The phylogenetic tree constructed based on PAVs is also similar to the phylogenetic tree based on SNPs (Figure 4B). These results suggest the PAVs are reliable. The numbers of genes in the core genome and pan-genome were simulated. Both of them reached their limit numbers before the number of genomes used in this study, which indicates the samples were sufficient for pan-genome construction and PAV analysis (Figure 4C).

**Figure 5:**
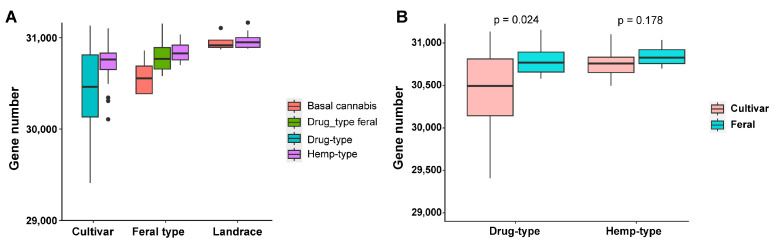
Number of genes in Cannabis samples. (**A**) The number of genes in different types of Cannabis samples. (**B**) The number of genes in drug types and hemp types; the colors represent wild varieties and cultivated varieties, respectively. Gene loss was observed during domestication.

To analyze the difference in gene numbers in different categories of Cannabis, PAVs were used to count gene numbers (Figure 5). The variety with the largest number of genes is NER2, which has 31,166 genes and is a cultivar of hemp-type Cannabis. The variety with the lowest number of genes is FNA, which has 28,174 genes and is a landrace. In cultivar and feral varieties, drug types have fewer genes than hemp types (Figure 5A). Compared with feral varieties, cultivar varieties have fewer genes, especially in drug-type Cannabis (Figure 5B). This shows significant gene loss during domestication and modern breeding. Gene loss is very common during domestication and modern breeding, and is found in tomato, soybean, and other pan-genomes.

**Figure 6:**
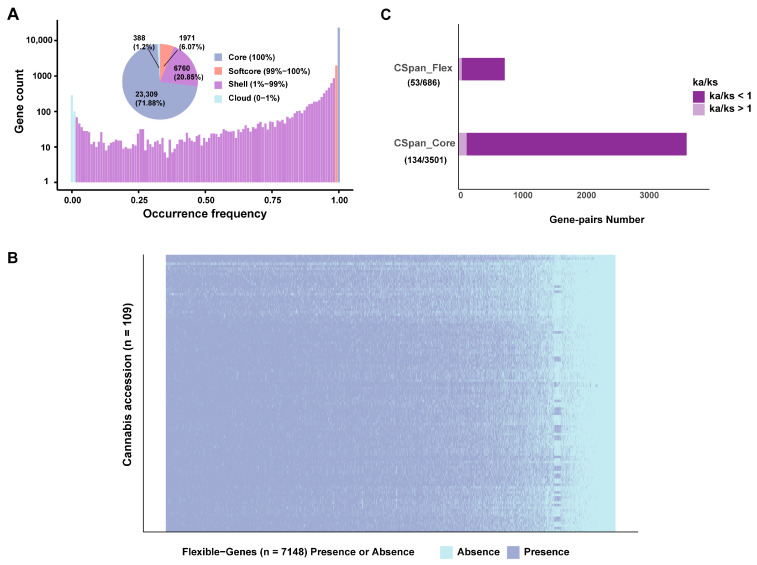
Pan-genome of *Cannabis sativa.* (**A**) Gene number and presence frequency in *C. sativa* pan genes. The pie chart corresponds to the core (present in all accessions), softcore, shell, and cloud genes. (**B**) A total of 109 *Cannabis sativa* accessions; heatmap shows presence and absence of flexible PAVs. (**C**) Ratio of nonsynonymous/synonymous (Ka/Ks) mutations of core and flexible genes.

The Cannabis pan-genome was divided into four parts, namely core genes (contained in 100% of varieties), softcore genes (contained in 99–100% of varieties), shell genes (contained in 1–99% of varieties), and cloud genes (contained in 0–1% of varieties). A total of 32,428 genes were detected in the Cannabis pan-genome PAV analysis, of which 32,140 genes were successfully identified, accounting for 99.11% of all genes. In this pan-genome, there are a total of 23,309 core genes (71.88% of the pan-genome), 1971 softcore genes (6.07%), 6760 shell genes (20.85%), and 388 cloud genes (1.2%) (Figure 6A,B). In the core genes, a total of 3719 gene pairs were obtained, of which 134 gene pairs (3.83%) had Ka/Ks (the number of nonsynonymous substitutions per nonsynonymous site/the number of synonymous substitutions per synonymous site) greater than 1, and 3501 gene pairs had Ka/Ks less than 1. In the flexible genes (shell genes and cloud genes), a total of 739 gene pairs were obtained, of which 53 gene pairs (7.17%) had Ka/Ks greater than 1, and 686 gene pairs had Ka/Ks less than 1. In these two groups, the proportion of gene pairs with Ka/Ks > 1 in flexible genes was significantly higher (Chi-squared *p* value < 2.2 × 10^−16^), indicating that a higher proportion of flexible genes exhibited signals of positive selection, likely reflecting their involvement in adaptive processes (Figure 6C).

**Figure 7:**
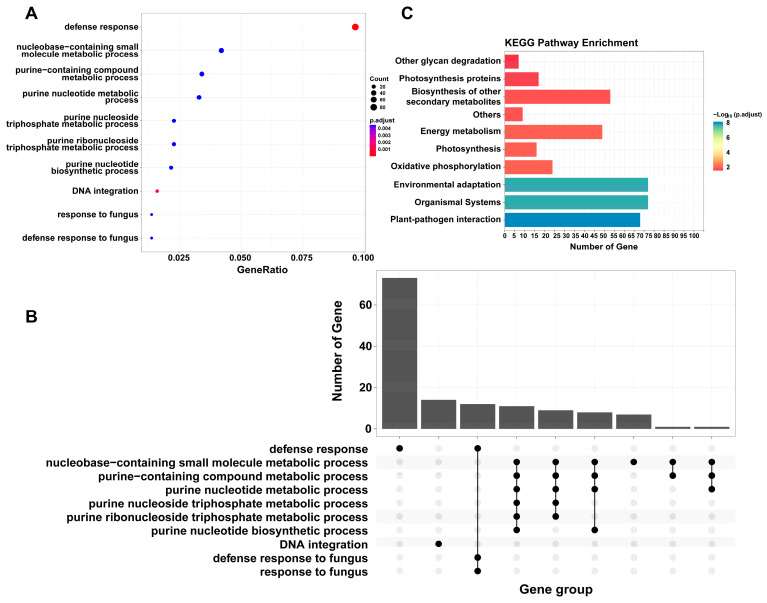
GO enrichment analysis of flexible genes. (**A**) Dotplot of flexible genes (BP). (**B**) Upsetplot of flexible genes (BP). (**C**) KEGG enrichment analysis of flexible genes. The functions of flexible genes are mainly related to adaption to the environment.

To better understand the differences between flexible genes and core genes, GO enrichment analysis and KEGG enrichment analysis were performed on these two gene sets. Core genes were only enriched in the GO terms related to basic activities of life, including “hydrolase activity”, “pyrophosphatase activity”, and “ubiquitin-protein transferase activity”. In contrast, flexible genes were enriched in “defense response”, “DNA integration”, “response to fungi”, “defense response to fungi”, and various metabolic processes and purine biosynthesis processes (Figure 7A,B). Among them, the largest number of genes were enriched in the “defense response” entry, which includes “defense response to fungus” and “response to fungus” (Figure 7B). Six GO terms were related to purine nucleotide metabolism, but the total gene number was much less than “defense response” (Figure 7B). This shows that flexible genomes are significantly related to adaptation to the environment.

To understand flexible genes’ function on the pathway level, KEGG pathway enrichment analysis was performed (Figure 7C). Flexible genes were mainly enriched in “Plant–pathogen interaction”; this shows that there are a large number of disease-resistant genes in the flexible genes, which may be the reason why Cannabis has strong disease resistance. Enriching in “Environmental adaptation” and “Biosynthesis of other secondary metabolites” shows these flexible genes are related to the abiotic stress resistance. Combined with the results of GO enrichment, we found that the flexible genes of Cannabis are mainly involved in defense, including biotic and abiotic stress resistance. This finding provides a genetic resource for Cannabis breeding and research, especially for resistance breeding.

## 3. Discussion

Genetic studies of crop domestication and improvement rely heavily on high-quality reference genome sequences of related plant species [32]. In this study, an iterative assembly method was used to construct the pan-genome using resequenced data from Cannabis varieties around the world and the reference genome of CBDRx [8], a high-quality hemp-type Cannabis variety, and the prediction of protein-encoding genes was completed (Table 2 and Table 3).

Retrotransposon LINEs have the potential to mediate nonallelic homologous recombination (NAHR) [21], which can result in long genomic DNA deletion or duplication [22,23]. LINEs present more in the Cannabis pan-genome than its reference genome (Table 1, Figure 1); this may explain the absence of non-reference sequences in the reference genome. This is not reported in pan-genome analysis and is worth focusing more attention on in future research. This inference also indicates genes beside LINEs may be lost from cultivated varieties, which can affect breeding strategy.

Population genetics and PAV analyses were performed using the Cannabis pan-genome. We investigated the genetic differentiation among Cannabis varieties and identified genes associated with the domestication process. Understanding the population structure and diversity of crop genomes will facilitate genetic manipulation, and will also enhance the plant breeding program [33]. We found that Cannabis varieties clearly clustered according to their types (Figure 2 and Figure 4), and only gene flow between the basal Cannabis group and feral drug-type Cannabis population was found (Figure 2C). The possible reason is that cultivated Cannabis populations are usually constituted by local varieties [34]. This indicates few gene communications happened between different types of Cannabis, so there is great potential for future Cannabis breeding by introducing genes from different types of varieties.

High genomic diversity was presented on SNP level (Figure 2) and gene level (Figure 4, Figure 5, Figure 6 and Figure 7), which provides rich materials for breeding and researching gene function and gene evolution. We found that 22.05% of the genes in Cannabis are flexible genes (Figure 6), lower than cotton (38%) [18], wild soybean (51%) [35], and rice (69.4%) [17] and higher than tomato (17.9%) [36]. Similarly to these pan-genomes, gene loss during domestication was observed, which shows gene loss is very common during domestication; however, gene gain during domestication was observed in the apple pan-genome [24]. For breeding, gene loss indicates that the lost genes and flexible genes can be introduced into new cultivars to gain corresponding functions and traits.

PAV analysis shows that flexible genes in Cannabis are mainly associated with environment adaption and disease resistance (Figure 7); this is also consistent with other published pan-genomes [12,14,15,17,18,36]. The flexible genes and their distribution in varieties in this study are valuable in Cannabis breeding and many other studies.

## 4. Materials and Methods

### 4.1. Construction of Cannabis Pan-Genome

In this study, the genomes of three main cultivated varieties in Northeast China, Qing1 (Q1) and Qing2 (Q2) cultivated by the Daqing Branch of Heilongjiang Academy of Agricultural Sciences, and the newly improved variety Senxin1 (SX1), were sequenced. Genomic DNA was extracted using FastPure Plant DNA Isolation Mini Kit (Vazyme Biotech, Nanjing, China) from seedlings; libraries were constructed using TruePrep DNA Library Prep Kit (Vazyme Biotech) and were sequenced on Illumina NovaSeq6000. Sequencing data were combined with 110 points of Cannabis resequencing data from all over the world for pan-genome construction. The genome of CBDRx (GCF_900626175.2) was used as a reference genome.

The software Trimmomatic (v0.39) [37] was used to remove joints and filter low-quality fragments. The processed fastq files were mapped to the reference genome CBDRx using MiniMap2 [38], and then Samtools [39] was used to output the sequences that did not match the reference genome. Megahit [40] was used to assemble these non-reference sequences into contigs.

We used MMseqs2 [41] to cluster the non-reference sequences and remove redundancy, and re-compared the obtained sequences to the reference genome. Overlapping regions were merged using Bedtools [42]; sequences with lengths greater than 500 bp and overlapping rates with the reference genome of less than 80% were output. We merged the mitochondrial and chloroplast sequences of Cannabis [43] and used MMseqs2 and Cap3 [44] to cluster the non-reference sequence obtained in the previous step with similar sequences of mitochondria and chloroplasts. Then the sequences similar to the mitochondria and chloroplast genomes were removed. The rest of the sequences were aligned to NCBI’s nt database; sequences matched to non-plant accessions were deleted to remove contamination.

### 4.2. Repetitive Sequence Masking of Non-Reference Sequences

After obtaining the de-redundant non-reference sequence, the repetitive sequence needed to be masked. First, RepeatModeler [45] was used for de novo prediction of repeat sequences. RepeatMasker [46] was used with RepeatModeler’s results and Dfam3.7 and Repbase (v20181026) used to mask repetitive sequences. The statistics of the masked repetitive sequence information were completed in R. The masked non-reference sequences were merged with the reference genome to complete the construction of the Cannabis pan-genome.

### 4.3. Gene Prediction and Annotation of Cannabis Pan-Genome

The gene prediction of the non-reference sequence of the Cannabis pan-genome was completed by Augustus and MAKER3 [47]. Trinity [48] was used in advance to assemble Cannabis transcripts as evidence of transcripts for gene prediction. NCBI’s nr database and UniProt (Swiss-Prot, TrEMBL, Uniref50) databases were used for gene annotation. The protein domain, GO, etc., were annotated using Interproscan [49], and the corresponding GO ID was extracted from the results. The KEGG annotation was obtained by KofamKOALA [50].

### 4.4. Population Genetic Analysis Based on Cannabis Pan-Genome

MiniMap2 was used to map the resequenced data to the Cannabis pan-genome, and then Samtools was used to obtain the bam file. Samtools and Sambamba [51] were used to sort and de-duplicate bam files. BCFtools [52] was used for SNP calling and filtering. The filtering criteria were as follows: only bi-allelic SNPs, SNP quality value >10, more than 70% of the sample contained, and a minimum allele frequency greater than 0.05.

Referring to the previous research on the apple pan-genome [24], this study used SNP data to calculate the distance matrix, and then used the distance matrix to construct the evolutionary tree. First, VCF2Dis was used to calculate the distance matrix, then Fastme [53] was used to construct the phylogenetic tree, and the tree was visualized by iTOL [54]. Principal component analysis was carried out using Plink [55], and subsequent analysis was carried out using R. The visualization was completed by the R package ggplot2 (v3.5.0).

Allele frequencies were calculated using Plink, format conversion was performed by Plink2treemix.py (https://github.com/barbatom/plink2treemix, accessed on 12 March 2024), and gene flow analysis was performed using Treemix [56] (m-values were taken from 0 to 10, and each operation was repeated three times).

VCFtools [57] was used to calculate Fst. XP-CLR (https://github.com/hardingnj/xpclr, accessed on 12 March 2024, v1.1.2) was used to complete the calculation. The subsequent data analysis was performed by R, and the visualization was performed by the R package CMplot (https://github.com/YinLiLin/CMplot, accessed on 10 February 2024) and ggplot2. XP-CLR score and Fst were used to find out the selection signals in the pan-genome. The top 10% of XP-CLR scores and Fst values were screened out, and the common segments were used as the selection interval. The genes in the selection interval were extracted by script, the gene annotation and enrichment analysis in the selection interval were completed by R language and TBtools (v2.042) [58], and the visualization was completed in R.

### 4.5. PAV Analysis Based on Cannabis Pan-Genome

All the resequencing data were mapped to the pan-genome of Cannabis by Minimap2 to obtain the BAM files. The PAV identification was completed using Hupan [59] to invoke the gene prediction result and the previously obtained bam file, and geneExist (parameters: 0.4 0.4) was used to generate the PAV file.

### 4.6. Assessment of the Pan-Genome of Cannabis

Using R language scripts for modeling, the scale of the core genome and pan-genome in the Cannabis pan-genome were evaluated as the sample filling changed, and the type and sample of the Cannabis pan-genome were judged to be sufficient.

### 4.7. Statistics on the Number and Frequency of Genes in the Cannabis Pan-Genome

The genes that exist in each sample, that is, the genes with a presence rate of 100%, were divided into core genes, the genes with a presence rate of 99–100% in the sample were divided into softcore genes, the genes with a presence rate of 1–99% in the sample were divided into shell genes, and the genes with a presence rate of less than or equal to 1% in the sample were divided into cloud genes.

### 4.8. Principal Component Analysis and Phylogenetic Analysis of Cannabis Pan-Genome PAVs

The principal component analysis and visualization based on PAVs were all performed by ggplot2 (the prcomp function completes the calculation). IQtree2 [60] was used to construct the developmental tree (maximum likelihood method).

### 4.9. Analysis of Flexible Genes and Core Genes

The shell genes and cloud genes were merged as flexible genes. The Ka/Ks calculation of core genes and variable genes was completed by wgd [61]. First, Orthofinder [62] was used to identify homologous gene pairs, and then wgd was used to calculate Ka/Ks. Enrichment analysis and visualization of GO and KEGG pathway were performed by clusterProfiler [63] and Enrichplot. More levels of enrichment analysis were performed by TBtools.

## 5. Conclusions

We sequenced genomes of three Cannabis varieties and constructed the Cannabis pan-genome. Finally, we assembled 24,679,380 bp genome sequences outside the reference genome, and identified a total of 1313 protein-coding genes in it. A total of 13,905,272 SNPs were obtained based on the pan-genome. Phylogenetic analysis and principal component analysis showed that the sample grouping was significant, consistent with the previous research results. A total of 32,428 gene PAVs were obtained from 109 Cannabis samples, and 7148 flexible genes accounting for 22.05% of all genes in the pan-genome were identified. Flexible genes were mainly related to traits that adapted to the living environment, such as Cannabis resistance and disease resistance. This result provides an important genetic basis and guiding method for targeted Cannabis breeding.

## Figures and Tables

**Table 1:** Distribution of repeating sequences in non-reference sequences.

	Number	Length (bp)	Proportion
Retroelement	12,772	8,080,389	32.74%
DNA transposon	2138	783,571	3.18%
Rolling circles	272	81,427	0.33%
Unclassified	14,822	3,105,612	12.58%
Small RNA	73	28,829	0.12%
Satellites	86	16,393	0.07%
Simple repeats	8912	383,968	1.56%
Low complexity	2248	120,709	0.49%

**Table 2:** Gene number.

	Reference Genome	Non-Reference Sequences	Non-Reference Sequences (The Length of the Encoded Protein > 100 aa)
Gene number	31,170	1919	1313
mRNA number	33,639	1919	1313
Exon number	234,131	3619	2858
CDS number	190,424	3592	2840
CDS length (bp)	46,210,977	1,143,501	1,006,458

**Table 3:** Gene annotation.

	The Number of Sequences Annotated	Proportion of All Sequences
Swiss-Prot	26,868	75.49%
Trembl	35,168	98.81%
Uniref50	35,275	99.11%
NCBI_nr	35,198	98.89%
Interproscan	33,345	93.68%
GO	20,953	58.87%
KEGG	13,865	38.95%
Uniprot and GOA	33,402	93.84%
NCBI_nr and Uniprot	35,294	99.16%

## Data Availability

The genome sequencing data of Qing1, Qing2, and Senxin1 has been deposited in the Genome Sequence Archive in the National Genomics Data Center of China (GSA: CRA028873) and are publicly accessible at https://ngdc.cncb.ac.cn/gsa.

## References

[1] Salentijn E.M.J., Zhang Q., Amaducci S., Yang M., Trindade L.M. (2015). New developments in fiber hemp (*Cannabis sativa* L.) breeding. Ind. Crops Prod..

[2] Li H.-L. (1974). The origin and use of cannabis in eastern asia linguistic-cultural implications. Econ. Bot..

[3] Ren M., Tang Z., Wu X., Spengler R., Jiang H., Yang Y., Boivin N. (2019). The origins of cannabis smoking: Chemical residue evidence from the first millennium BCE in the Pamirs. Sci. Adv..

[4] Hofmann-Aßmus M. (2020). THC/CBD wirkt besser als THC allein. MMW—Fortschritte Med..

[5] Daris B., Tancer Verboten M., Knez Z., Ferk P. (2019). Cannabinoids in cancer treatment: Therapeutic potential and legislation. Bosn. J. Basic. Med. Sci..

[6] Kuzumi A., Yoshizaki-Ogawa A., Fukasawa T., Sato S., Yoshizaki A. (2024). The Potential Role of Cannabidiol in Cosmetic Dermatology: A Literature Review. Am. J. Clin. Dermatol..

[7] van Bakel H., Stout J.M., Cote A.G., Tallon C.M., Sharpe A.G., Hughes T.R., Page J.E. (2011). The draft genome and transcriptome of *Cannabis sativa*. Genome Biol..

[8] Grassa C.J., Weiblen G.D., Wenger J.P., Dabney C., Poplawski S.G., Timothy Motley S., Michael T.P., Schwartz C.J. (2021). A new Cannabis genome assembly associates elevated cannabidiol (CBD) with hemp introgressed into marijuana. New Phytol..

[9] Ren G., Zhang X., Li Y., Ridout K., Serrano-Serrano M.L., Yang Y., Liu A., Ravikanth G., Nawaz M.A., Mumtaz A.S. (2021). Large-scale whole-genome resequencing unravels the domestication history of *Cannabis sativa*. Sci. Adv..

[10] Bian P.P., Zhang Y., Jiang Y. (2021). Pan-genome: Setting a new standard for high-quality reference genomes. Yi Chuan.

[11] Song J.M., Guan Z., Hu J., Guo C., Yang Z., Wang S., Liu D., Wang B., Lu S., Zhou R. (2020). Eight high-quality genomes reveal pan-genome architecture and ecotype differentiation of *Brassica napus*. Nat. Plants.

[12] Zhang F., Xue H., Dong X., Li M., Zheng X., Li Z., Xu J., Wang W., Wei C. (2022). Long-read sequencing of 111 rice genomes reveals significantly larger pan-genomes. Genome Res..

[13] Wang W., Mauleon R., Hu Z., Chebotarov D., Tai S., Wu Z., Li M., Zheng T., Fuentes R.R., Zhang F. (2018). Genomic variation in 3,010 diverse accessions of Asian cultivated rice. Nature.

[14] Hubner S., Bercovich N., Todesco M., Mandel J.R., Odenheimer J., Ziegler E., Lee J.S., Baute G.J., Owens G.L., Grassa C.J. (2019). Sunflower pan-genome analysis shows that hybridization altered gene content and disease resistance. Nat. Plants.

[15] Liu Y., Du H., Li P., Shen Y., Peng H., Liu S., Zhou G.A., Zhang H., Liu Z., Shi M. (2020). Pan-Genome of Wild and Cultivated Soybeans. Cell.

[16] Cai X., Chang L., Zhang T., Chen H., Zhang L., Lin R., Liang J., Wu J., Freeling M., Wang X. (2021). Impacts of allopolyploidization and structural variation on intraspecific diversification in *Brassica rapa*. Genome Biol..

[17] Qin P., Lu H., Du H., Wang H., Chen W., Chen Z., He Q., Ou S., Zhang H., Li X. (2021). Pan-genome analysis of 33 genetically diverse rice accessions reveals hidden genomic variations. Cell.

[18] Li J., Yuan D., Wang P., Wang Q., Sun M., Liu Z., Si H., Xu Z., Ma Y., Zhang B. (2021). Cotton pan-genome retrieves the lost sequences and genes during domestication and selection. Genome Biol..

[19] Dolatabadian A., Bayer P.E., Tirnaz S., Hurgobin B., Edwards D., Batley J. (2020). Characterization of disease resistance genes in the *Brassica napus* pangenome reveals significant structural variation. Plant Biotechnol. J..

[20] Benedetti E., Resce G., Brunori P., Molinaro S. (2021). Cannabis Policy Changes and Adolescent Cannabis Use: Evidence from Europe. Int. J. Environ. Res. Public Health.

[21] Startek M., Szafranski P., Gambin T., Campbell I.M., Hixson P., Shaw C.A., Stankiewicz P., Gambin A. (2015). Genome-wide analyses of LINE-LINE-mediated nonallelic homologous recombination. Nucleic Acids Res..

[22] Dittwald P., Gambin T., Szafranski P., Li J., Amato S., Divon M.Y., Rodriguez Rojas L.X., Elton L.E., Scott D.A., Schaaf C.P. (2013). NAHR-mediated copy-number variants in a clinical population: Mechanistic insights into both genomic disorders and Mendelizing traits. Genome Res..

[23] Stankiewicz P., Lupski J.R. (2002). Genome architecture, rearrangements and genomic disorders. Trends Genet..

[24] Sun X., Jiao C., Schwaninger H., Chao C.T., Ma Y., Duan N., Khan A., Ban S., Xu K., Cheng L. (2020). Phased diploid genome assemblies and pan-genomes provide insights into the genetic history of apple domestication. Nat. Genet..

[25] Sun X., Zhang Z., Wu J., Cui X., Feng D., Wang K., Xu M., Zhou L., Han X., Gu X. (2016). The *Oryza sativa* Regulator HDR1 Associates with the Kinase OsK4 to Control Photoperiodic Flowering. PLoS Genet..

[26] Sun A., Yu B., Zhang Q., Peng Y., Yang J., Sun Y., Qin P., Jia T., Smeekens S., Teng S. (2020). MYC2-Activated TRICHOME BIREFRINGENCE-LIKE37 Acetylates Cell Walls and Enhances Herbivore Resistance. Plant Physiol..

[27] Liu S., Chen S., Zhou Y., Shen Y., Qin Z., Wu L. (2023). VERNALIZATION1 represses FLOWERING PROMOTING FACTOR1-LIKE1 in leaves for timely flowering in *Brachypodium distachyon*. Plant Cell.

[28] Taurino M., Costantini S., De Domenico S., Stefanelli F., Ruano G., Delgadillo M.O., Sanchez-Serrano J.J., Sanmartin M., Santino A., Rojo E. (2018). SEIPIN Proteins Mediate Lipid Droplet Biogenesis to Promote Pollen Transmission and Reduce Seed Dormancy. Plant Physiol..

[29] Wang X., Chai X., Gao B., Deng C., Gunther C.S., Wu T., Zhang X., Xu X., Han Z., Wang Y. (2023). Multi-omics analysis reveals the mechanism of bHLH130 responding to low-nitrogen stress of apple rootstock. Plant Physiol..

[30] Weingartner M., Subert C., Sauer N. (2011). LATE, a C(2)H(2) zinc-finger protein that acts as floral repressor. Plant J..

[31] Cheng Q., Tong Y., Wang Z., Su P., Gao W., Huang L. (2017). Molecular cloning and functional identification of a cDNA encoding 4-hydroxy-3-methylbut-2-enyl diphosphate reductase from *Tripterygium wilfordii*. Acta Pharm. Sin. B.

[32] Huang X., Huang S., Han B., Li J. (2022). The integrated genomics of crop domestication and breeding. Cell.

[33] Ingvardsen C.R., Brinch-Pedersen H. (2023). Challenges and potentials of new breeding techniques in Cannabis sativa. Front. Plant Sci..

[34] Barcaccia G., Palumbo F., Scariolo F., Vannozzi A., Borin M., Bona S. (2020). Potentials and Challenges of Genomics for Breeding Cannabis Cultivars. Front. Plant Sci..

[35] Li Y.H., Zhou G., Ma J., Jiang W., Jin L.G., Zhang Z., Guo Y., Zhang J., Sui Y., Zheng L. (2014). De novo assembly of soybean wild relatives for pan-genome analysis of diversity and agronomic traits. Nat. Biotechnol..

[36] Gao L., Gonda I., Sun H., Ma Q., Bao K., Tieman D.M., Burzynski-Chang E.A., Fish T.L., Stromberg K.A., Sacks G.L. (2019). The tomato pan-genome uncovers new genes and a rare allele regulating fruit flavor. Nat. Genet..

[37] Bolger A.M., Lohse M., Usadel B. (2014). Trimmomatic: A flexible trimmer for Illumina sequence data. Bioinformatics.

[38] Li H. (2021). New strategies to improve minimap2 alignment accuracy. Bioinformatics.

[39] Li H., Handsaker B., Wysoker A., Fennell T., Ruan J., Homer N., Marth G., Abecasis G., Durbin R., 1000 Genome Project Data Processing Subgroup (2009). The Sequence Alignment/Map format and SAMtools. Bioinformatics.

[40] Li D., Liu C.M., Luo R., Sadakane K., Lam T.W. (2015). MEGAHIT: An ultra-fast single-node solution for large and complex metagenomics assembly via succinct *de Bruijn* graph. Bioinformatics.

[41] Steinegger M., Soding J. (2017). MMseqs2 enables sensitive protein sequence searching for the analysis of massive data sets. Nat. Biotechnol..

[42] Quinlan A.R., Hall I.M. (2010). BEDTools: A flexible suite of utilities for comparing genomic features. Bioinformatics.

[43] Vergara D., White K.H., Keepers K.G., Kane N.C. (2016). The complete chloroplast genomes of *Cannabis sativa* and *Humulus lupulus*. Mitochondrial DNA A DNA Mapp. Seq. Anal..

[44] Huang X., Madan A. (1999). CAP3: A DNA sequence assembly program. Genome Res..

[45] Flynn J.M., Hubley R., Goubert C., Rosen J., Clark A.G., Feschotte C., Smit A.F. (2020). RepeatModeler2 for automated genomic discovery of transposable element families. Proc. Natl. Acad. Sci. USA.

[46] Chen N. (2004). Using RepeatMasker to identify repetitive elements in genomic sequences. Curr. Protoc. Bioinform..

[47] Cantarel B.L., Korf I., Robb S.M., Parra G., Ross E., Moore B., Holt C., Sanchez Alvarado A., Yandell M. (2008). MAKER: An easy-to-use annotation pipeline designed for emerging model organism genomes. Genome Res..

[48] Grabherr M.G., Haas B.J., Yassour M., Levin J.Z., Thompson D.A., Amit I., Adiconis X., Fan L., Raychowdhury R., Zeng Q. (2011). Full-length transcriptome assembly from RNA-Seq data without a reference genome. Nat. Biotechnol..

[49] Jones P., Binns D., Chang H.Y., Fraser M., Li W., McAnulla C., McWilliam H., Maslen J., Mitchell A., Nuka G. (2014). InterProScan 5: Genome-scale protein function classification. Bioinformatics.

[50] Aramaki T., Blanc-Mathieu R., Endo H., Ohkubo K., Kanehisa M., Goto S., Ogata H. (2020). KofamKOALA: KEGG Ortholog assignment based on profile HMM and adaptive score threshold. Bioinformatics.

[51] Tarasov A., Vilella A.J., Cuppen E., Nijman I.J., Prins P. (2015). Sambamba: Fast processing of NGS alignment formats. Bioinformatics.

[52] Danecek P., Bonfield J.K., Liddle J., Marshall J., Ohan V., Pollard M.O., Whitwham A., Keane T., McCarthy S.A., Davies R.M. (2021). Twelve years of SAMtools and BCFtools. Gigascience.

[53] Lefort V., Desper R., Gascuel O. (2015). FastME 2.0: A Comprehensive, Accurate, and Fast Distance-Based Phylogeny Inference Program. Mol. Biol. Evol..

[54] Letunic I., Bork P. (2021). Interactive Tree Of Life (iTOL) v5: An online tool for phylogenetic tree display and annotation. Nucleic Acids Res..

[55] Purcell S., Neale B., Todd-Brown K., Thomas L., Ferreira M.A., Bender D., Maller J., Sklar P., de Bakker P.I., Daly M.J. (2007). PLINK: A tool set for whole-genome association and population-based linkage analyses. Am. J. Hum. Genet..

[56] Pickrell J.K., Pritchard J.K. (2012). Inference of population splits and mixtures from genome-wide allele frequency data. PLoS Genet..

[57] Danecek P., Auton A., Abecasis G., Albers C.A., Banks E., DePristo M.A., Handsaker R.E., Lunter G., Marth G.T., Sherry S.T. (2011). The variant call format and VCFtools. Bioinformatics.

[58] Chen C., Chen H., Zhang Y., Thomas H.R., Frank M.H., He Y., Xia R. (2020). TBtools: An Integrative Toolkit Developed for Interactive Analyses of Big Biological Data. Mol. Plant.

[59] Duan Z., Qiao Y., Lu J., Lu H., Zhang W., Yan F., Sun C., Hu Z., Zhang Z., Li G. (2019). HUPAN: A pan-genome analysis pipeline for human genomes. Genome Biol..

[60] Minh B.Q., Schmidt H.A., Chernomor O., Schrempf D., Woodhams M.D., von Haeseler A., Lanfear R. (2020). IQ-TREE 2: New Models and Efficient Methods for Phylogenetic Inference in the Genomic Era. Mol. Biol. Evol..

[61] Zwaenepoel A., Van de Peer Y. (2019). wgd-simple command line tools for the analysis of ancient whole-genome duplications. Bioinformatics.

[62] Emms D.M., Kelly S. (2019). OrthoFinder: Phylogenetic orthology inference for comparative genomics. Genome Biol..

[63] Yu G., Wang L.G., Han Y., He Q.Y. (2012). clusterProfiler: An R package for comparing biological themes among gene clusters. OMICS.

